# Bioinformatics Identification of Ferroptosis-Related Biomarkers and Therapeutic Compounds in Ischemic Stroke

**DOI:** 10.3389/fneur.2021.745240

**Published:** 2021-10-11

**Authors:** Guozhong Chen, Lin Li, Hongmiao Tao

**Affiliations:** ^1^School of Public Health, Hangzhou Normal University, Hangzhou, China; ^2^College of Basic Medical Sciences, Zhejiang Chinese Medical University, Hangzhou, China; ^3^Medical College, Jinhua Polytechnic, Jinhua, China

**Keywords:** ferroptosis, ischemic stroke, diagnostic biomarker, MAP1LC3B, PTGS2, TLR4

## Abstract

**Background:** Stroke is one of the most common deadly diseases with an estimated 780,000 new cases globally, of which ischemic stroke accounts for over 80% of all cases. Ferroptosis is a new form of programmed cell death that plays a vital role in many diseases, including ischemic stroke and heart diseases. The role of the ferroptosis-related gene in the diagnosis, prognosis, or therapy of ischemic stroke was not fully clarified.

**Methods:** Ferroptosis-related differentially expressed genes (DEGs) in ischemic stroke were identified by bioinformatic analysis of the GSE16561 and GSE22255 datasets. Subsequently, receiver operator characteristic (ROC) monofactor analysis was performed to evaluate the diagnostic value of ferroptosis-related biomarkers in ischemic stroke.

**Results:** A total of 10 ferroptosis-related DEGs were identified in ischemic stroke vs. normal control. GO and KEGG analysis revealed that these 10 ferroptosis-related DEGs were mainly enriched in response to oxidative stress, HIF-1 signaling pathway, ferroptosis, lipid, and atherosclerosis. Moreover, the random forest model suggested three ferroptosis-related biomarkers, namely, PTGS2, MAP1LC3B, and TLR4, for ischemic stroke. Interestingly, the expression of PTGS2, MAP1LC3B, and TLR4 was upregulated in ischemic stroke. ROC monofactor analysis demonstrated a good performance of MAP1LC3B, PTGS2, and TLR4 in the diagnosis of ischemic stroke. The expression and diagnostic value of MAP1LC3B, PTGS2, and TLR4 in ischemic stroke were also verified using GSE22255. We also revealed the transcription factor regulation network and co-expressed protein network of ferroptosis-related biomarkers. Several potential therapeutic compounds corresponding to MAP1LC3B, PTGS2, and TLR4 were also identified for ischemic stroke, including Zinc12503187 (Conivaptan), Zinc3932831 (Avodart), Zinc64033452 (Lumacaftor), Zinc11679756 (Eltrombopag), Zinc100378061 (Naldemedine), and Zinc3978005 (Dihydroergotamine).

**Conclusion:** Our results suggested MAP1LC3B, PTGS2, and TLR4 as potential diagnostic biomarkers for ischemic stroke, providing more evidence about the vital role of ferroptosis in ischemic stroke.

## Introduction

Stroke is one of the most common deadly diseases with an estimated 780,000 new cases globally, of which ischemic stroke accounts for over 80% of all cases (1, 2). Stroke is considered the second leading cause of death after ischemic heart disease and the third leading cause of disability worldwide (3). Once a stroke occurs, patients need to follow up and take medication for a long time, causing a huge financial, mental, and time burden. Though some risk factors, including hypertension, diabetes, hyperlipidemia, and smoking had been identified for ischemic stroke, the specific molecular mechanism of ischemic stroke is not fully clarified. Accumulating evidence revealed that early diagnosis of ischemic stroke can improve the therapy effect and prognosis of patients (4). These data suggested the urgency and importance of elucidation of potential mechanism of ischemic stroke, thus identifying novel biomarkers and therapeutic targets.

Ferroptosis is a new form of programmed cell death characterized by abundant iron accumulation and lipid peroxidation during the cell death process (5). Recent evidence revealed that ferroptosis was involved in the tumorigenesis and progression of cancer (6–8). Interestingly, ferroptosis was considered to play a vital role in many diseases, including ischemic stroke and heart diseases (9, 10). Moreover, ferroptosis-related gene signature could serve as a biomarker for the diagnosis, prognosis, or therapy for many cancers or disease (11, 12). However, the role of ferroptosis-related gene in the diagnosis, prognosis, or therapy of ischemic stroke was not fully clarified.

The development of molecular biology and next-generation sequencing technology makes it possible to explore the potential mechanism of diseases on a large scale at the genetic and mRNA level (13). By comparing the disease cohort and normal control cohort, we could obtain a sea of gene expression profiles and identify differentially expressed genes (DEGs) correlated with the generation and progression of disease, including ischemic stroke. In the current study, we isolated the ferroptosis-related DEGs between ischemic stroke and normal control cohort using GSE16561 and GSE22255 datasets. Receiver operator characteristic (ROC) monofactor analysis was performed to evaluate the diagnostic value of ferroptosis-related biomarkers in ischemic stroke. The results of our study may provide a molecular theoretical foundation for the development of diagnostic and therapy biomarkers.

## Materials and Methods

### Datasets and Pre-processing

Two ischemic stroke-related microarray datasets of GSE16561 and GSE22255 and transcriptome data of GSE140275 were downloaded from the GEO database, which is shown in Table 1.

**Table 1:** Ischemic stroke-related dataset from the GEO database.

**Dataset ID**	**Platform**	**Stroke**	**Normal**
GSE16561	GPL6883	39	24
GSE22255	GPL570	20	20
GSE140275	GPL16791	3	3

The data processing was performed using R project (R version 4.0.5) as follows: firstly, the probes were mapped to the gene and no-load probes were removed. If multiple probes were mapped to the same gene, the mean value was selected as the expression level of the gene. Gene expression was normalized to Log2-transformed quantile-normalized signal intensity for further analysis. The DEGs were identified using LIMMA package with *p*-value < 0.05 and a fold change of 2 as the threshold. It is worth noting that the removeBatcheffect function in the LIMMA package was used to eliminate the influence of gender on gene expression.

### Screening Ferroptosis-Related Biomarkers

A total of 192 ferroptosis-related biomarkers were obtained from the FerrDb database (http://www.zhounan.org/ferrdb/) and the latest ferroptosis-related study on May 4, 2021 (14, 15). The union sets of DEGs in GSE16561 and GSE140275 datasets were selected as candidate ferroptosis-related biomarkers for further analysis. Kyoto Encyclopedia of Genes and Genomes (KEGG) pathway and Gene Ontology (GO) analysis, including the biological process (BP), cellular component (CC), and molecular function (MF), were conducted with the “ggplot2” package in R software. Random-forest model was used to evaluate the importance of candidate ferroptosis-related biomarkers in GSE16561 and GSE140275 datasets, respectively. Moreover, 40% of all biomarkers with weak importance were filtered out according to the Gini index, and 60% of all biomarkers was retained. Ferroptosis-related biomarkers were identified by taking union sets of biomarkers retained above in GSE16561 and GSE140275 datasets.

### The Clinical Characteristics Difference and Diagnostic Value of Ferroptosis-Related Biomarkers in Ischemic Stroke

ROC monofactor analysis was performed in different datasets to evaluate the diagnostic value of ferroptosis-related biomarkers in ischemic stroke. The expression of ferroptosis-related biomarkers in different ages and genders of ischemic stroke patients was analyzed with Student's *t*-test. It is worth noting that GSE16561 and GSE140275 were used as the test dataset and GSE22255 was used as the verification dataset.

### Construction of a Multi-Factor Regulation Network

The transcription factors corresponding to ferroptosis-related biomarkers in ischemic stroke were isolated from the TRRUST v2 (https://www.grnpedia.org/trrust) database. The regulation network between transcription factors and ferroptosis-related biomarkers was drawn using the core transcription factors that could regulate multiple ferroptosis-related biomarkers. STRING (https://string-db.org/) was used to identify the proteins co-expressed with ferroptosis-related biomarkers with an interaction score (0.4). The network map was drawn based on these proteins. Moreover, GO and KEGG analyses were performed using the genes of co-expressed proteins.

Protein structure was isolated from the Protein Data Bank (PDB) database (https://www.rcsb.org/). FDA-approved small-molecule formulations were downloaded from the Zinc15 database (http://zinc.docking.org/). Molecular docking was performed by Autodock-Vina software, and the results were visualized using Discovery Studio software.

## Results

### Identification of DEGs and Ferroptosis-Related DEGs

**Figure 1:**
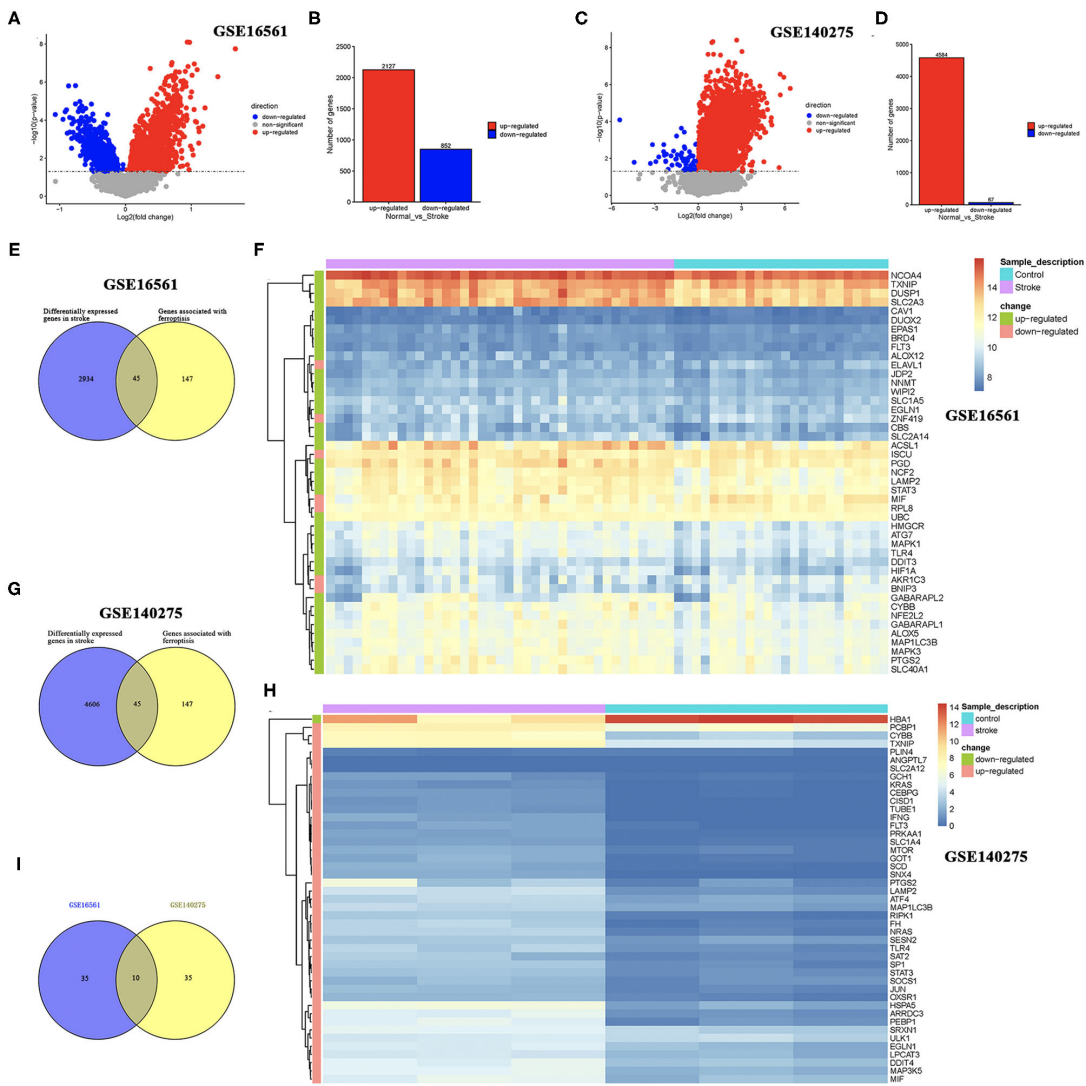
Identification of DEGs and ferroptosis-related DEGs. **(A,B)** The DEGs between ischemic stroke cohort and normal control cohort in the GSE16561 dataset. **(C,D)** The DEGs between ischemic stroke cohort and normal control cohort in the GSE140275 dataset. **(E,F)** The ferroptosis-related DEGs between ischemic stroke cohort and normal control cohort in the GSE16561 dataset. **(G,H)** The ferroptosis-related DEGs between ischemic stroke cohort and normal control cohort in the GSE140275 dataset. **(I)** The intersection of ferroptosis-related DEGs in the GSE16561 and GSE140275 datasets. DEGs, differentially expressed genes.

Two sets of gene expression data, GSE16561 and GSE140275, were downloaded from GEO, and the LIMMA package in R was used to analyze the difference between ischemic stroke and healthy controls in these two datasets with *p*-value < 0.05 as the threshold. As a result, a total of 2,127 upregulated genes and 852 downregulated genes were obtained from the GSE16561 dataset (Figures 1A,B). In the GSE140275 dataset, we isolated 4,584 upregulated genes and 67 downregulated genes (Figures 1C,D). A total of 192 ferroptosis-related genes were obtained from the FerrDb database (http://www.zhounan.org/ferrdb/) and the latest ferroptosis-related study (14, 15). Combining the results of 192 ferroptosis-related genes and DEGs in the GSE16561 and GSE140275 datasets, we obtained ferroptosis-related DEGs. As a result, 45 ferroptosis-related DEGs were identified in the GSE16561 dataset, among which only 7 genes were downregulated, accounting for 15.56%, and 38 genes were upregulated in ischemic stroke patients compared with the healthy control (Figures 1E,F). Interestingly, we also identified 45 ferroptosis-related DEGs in the GSE140275 dataset, among which only one gene was downregulated and 44 genes were upregulated (Figures 1G,H). Combining the ferroptosis-related DEGs of the GSE16561 and GSE140275 datasets, we finally obtained 10 candidate ferroptosis-related biomarkers (Figure 1I).

### GO and KEGG Analysis With Candidate Ferroptosis-Related Biomarkers

**Figure 2:**
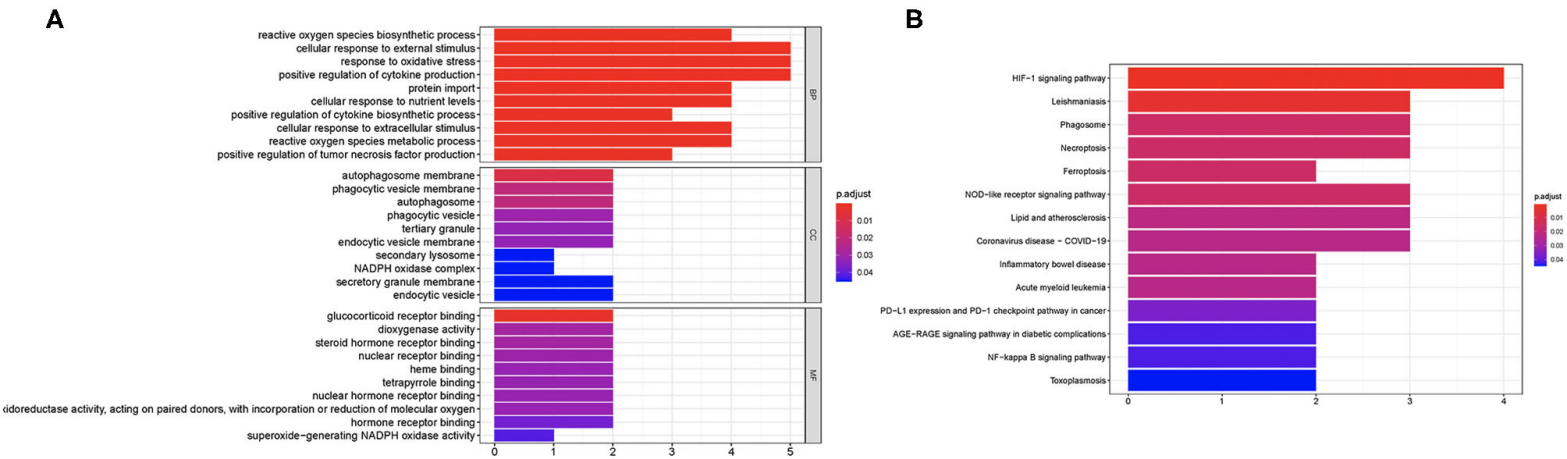
Enriched items in GO and KEGG analysis using ferroptosis-related DEGs. **(A)** The enriched items in GO analysis. **(B)** The enriched items in KEGG pathway analysis. DEGs, differentially expressed genes; GO, Gene Ontology; BP, biological process; CC, cellular component; MF, molecular function; KEGG, Kyoto Encyclopedia of Genes and Genomes.

We then performed GO and KEGG analysis using 10 candidate ferroptosis-related biomarkers. The result suggested that these candidate ferroptosis-related biomarkers were mainly enriched in cellular response to external stimulus, response to oxidative stress, and positive regulation of cytokine production in GO analysis (Figure 2A). Moreover, the results of KEGG revealed that these ferroptosis-related biomarkers were mainly enriched in the HIF-1 signaling pathway, Leishmaniasis, Phagosome, Necroptosis, Ferroptosis, NOD-like receptor signaling pathway, Lipid and atherosclerosis, and Coronavirus disease-COVID-19 (Figure 2B).

### Correlation Analysis and Random Forest Screening of Candidate Ferroptosis-Related Biomarkers

**Figure 3:**
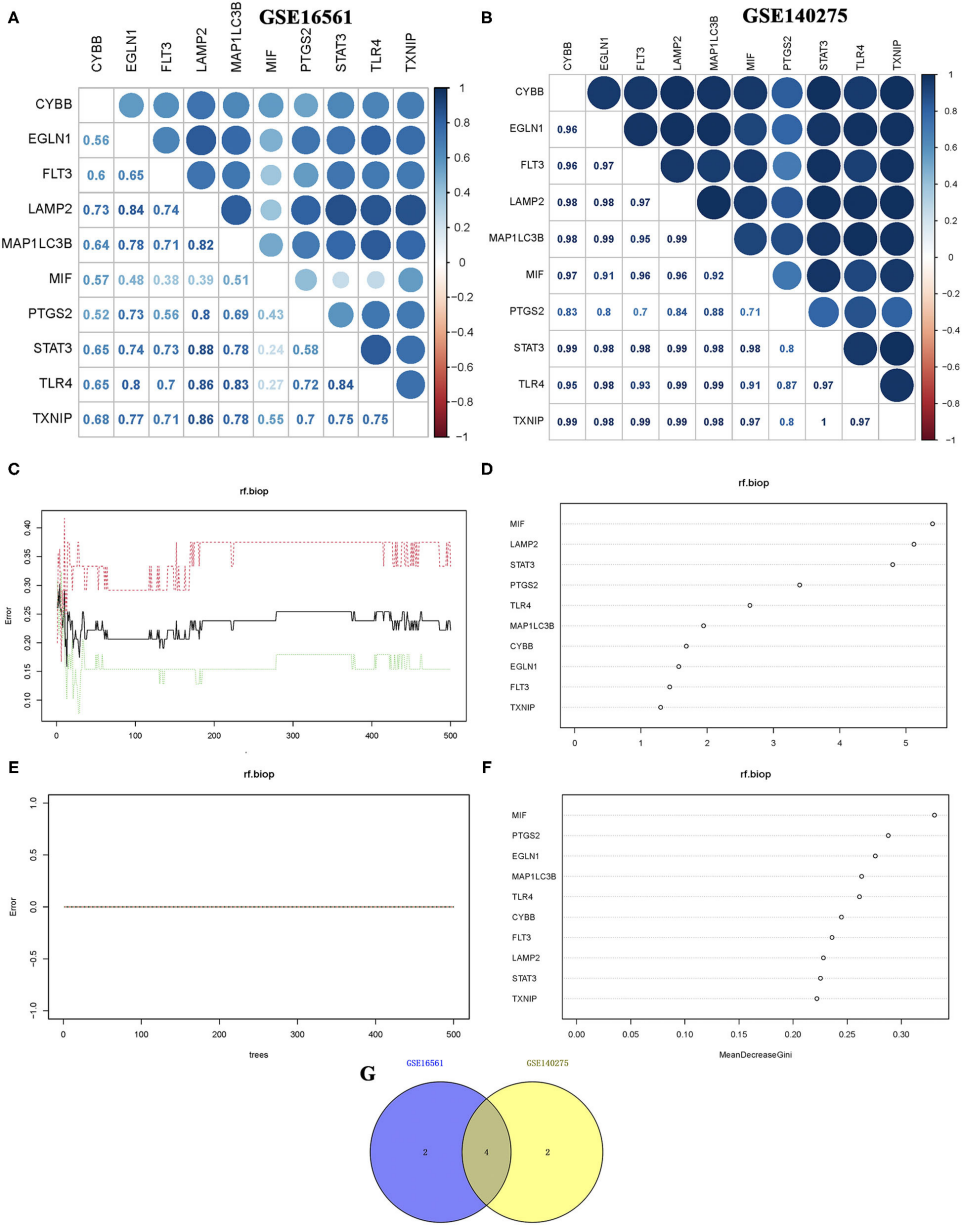
Correlation analysis and random forest screening of candidate ferroptosis-related biomarkers. **(A,B)** Correlation analysis of candidate ferroptosis-related biomarkers in GSE16561 and GSE140275 datasets. **(C,D)** Random forest screening of candidate ferroptosis-related biomarkers in the GSE16561 dataset. **(E,F)** Random forest screening of candidate ferroptosis-related biomarkers in the GSE140275 dataset. **(G)** Venn diagram revealed the ferroptosis-related biomarkers screening random forest analysis in GSE16561 and GSE140275 datasets.

Correlation analysis of candidate ferroptosis-related biomarkers showed significant positive correlation in GSE16561 (Figure 3A) and GSE140275 (Figure 3B) datasets, suggesting that these candidate ferroptosis-related biomarkers had synergistic interaction on expression. The random forest model was used to conduct deep learning on the sample data of GSE16561 and GSE140275. According to the Gini index of each candidate ferroptosis-related biomarker, 40% of all candidate ferroptosis-related biomarkers with weak importance were filtered out and 60% were retained. In GSE16561, the final prediction accuracy of the random forest model was 77.78%. The important ferroptosis-related biomarkers were MIF, LAMP2, STAT3, PTGS2, TLR4, and MAP1LC3B (Figures 3C,D). In GSE140275, the final prediction accuracy of the random forest model was 100%, and the important ferroptosis-related biomarkers were MIF, PTGS2, EGLN1, MAP1LC3B, TLR4, and CYBB (Figures 3E,F). A total of four ferroptosis-related biomarkers were obtained by taking intersection of these two datasets, which were MIF, PTGS2, MAP1LC3B, and TLR4 (Figure 3G).

### The Expression of Ferroptosis-Related Biomarkers in Ischemic Stroke

**Figure 4:**
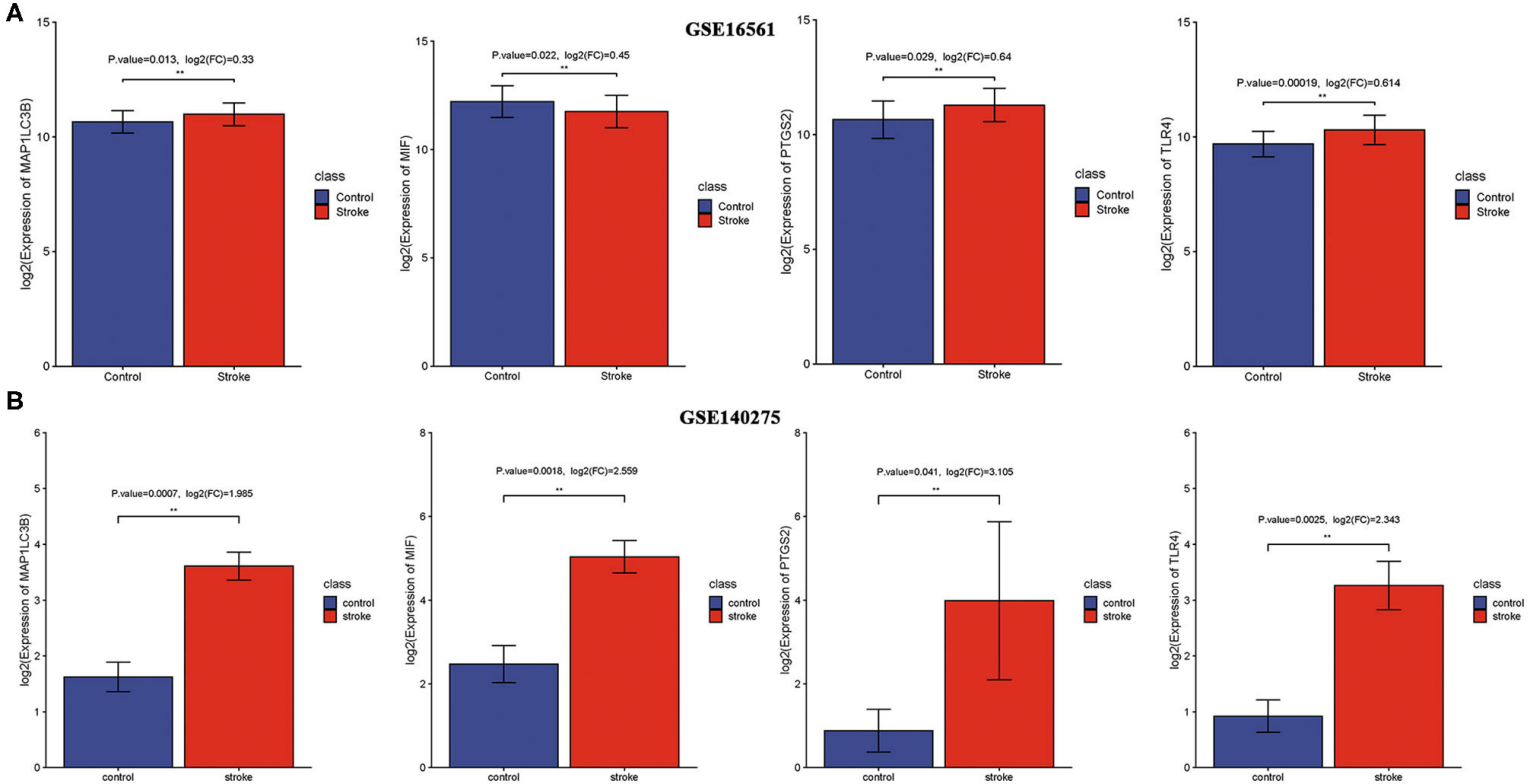
The expression of ferroptosis-related biomarkers in ischemic stroke. **(A)** The expression of ferroptosis-related biomarkers in ischemic stroke vs. normal control cohort in the GSE16561 dataset. **(B)** The expression of ferroptosis-related biomarkers in ischemic stroke vs. normal control cohort in the GSE140275 dataset. **p < 0.01.

We then detected the expression of these four ferroptosis-related biomarkers in ischemic stroke patients and normal control. The results revealed that MIF expression was downregulated in ischemic stroke patients vs. normal control in the GSE16561 dataset (*p* = 0.022, Figure 4A), while it was upregulated in the GSE140275 dataset (*p* = 0.0018, Figure 4B). The expression of three other ferroptosis-related biomarkers, PTGS2, MAP1LC3B, and TLR4, was all upregulated in ischemic stroke patients vs. normal control in the GSE16561 (Figure 4A, all *p* < 0.05) and GSE140275 datasets (Figure 4B, all *p* < 0.05). Finally, the non-cooperatively expressed MIF was removed, and PTGS2, MAP1LC3B, and TLR4 were further selected as ferroptosis-related biomarkers for ischemic stroke.

### The Expression of Ferroptosis-Related Biomarkers in a Subgroup of Ischemic Stroke Patients

**Figure 5:**
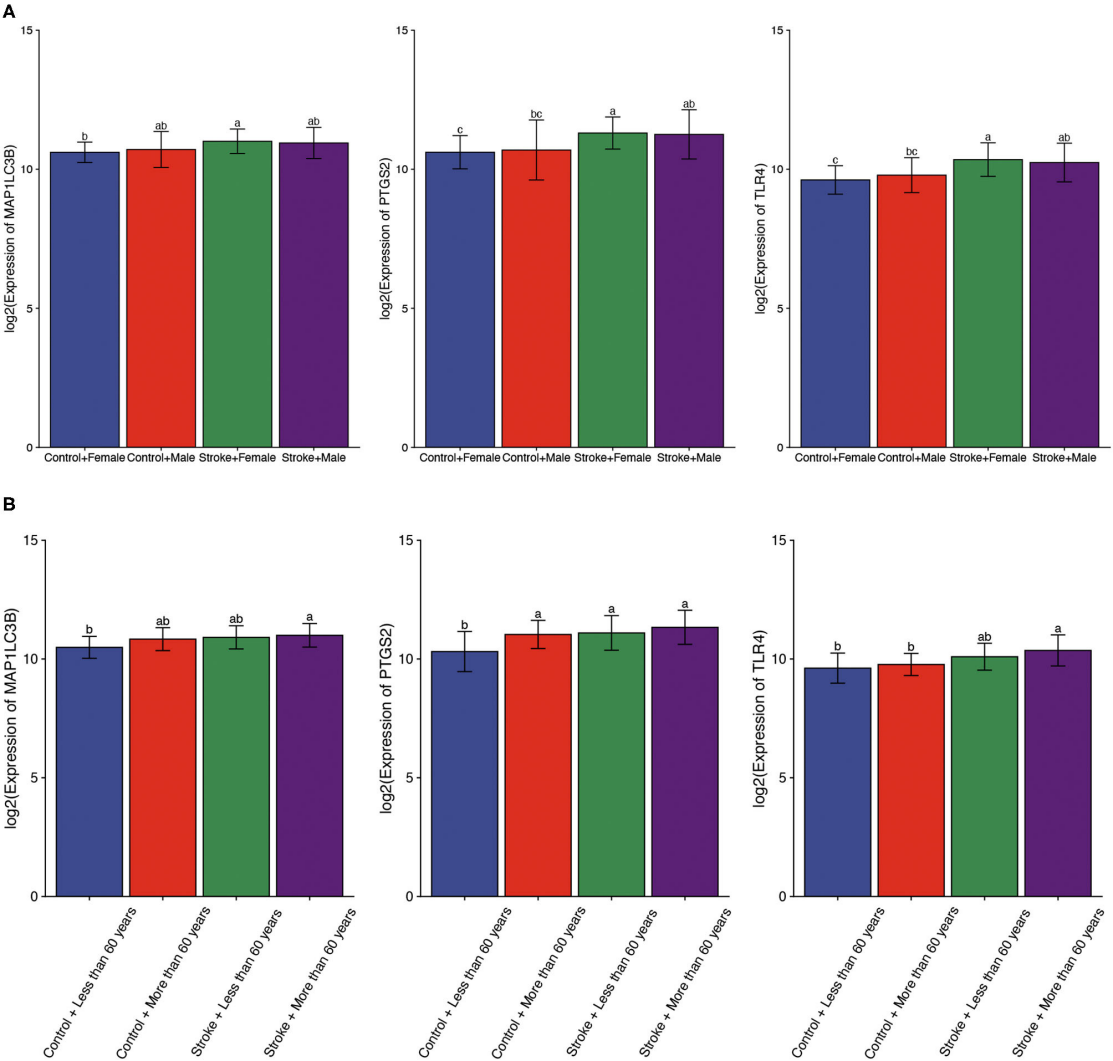
The expression of ferroptosis-related biomarkers in a subgroup of ischemic stroke. **(A)** The expression of ferroptosis-related biomarkers in different genders of ischemic stroke patients. **(B)** The expression of ferroptosis-related biomarkers in different ages of ischemic stroke patients. a, variance variance; b, variance analysis; c, common sense.

Using gender as a grouping variable in the GSE16561 dataset, no significant gender difference was obtained between ischemic stroke samples and normal control samples (Figure 5A). We then divided all cohort into two groups with the age of 60 as the cutoff value. As expected, the results showed that there was no significant difference in the expression of ferroptosis-related biomarkers between different ages (Figure 5B).

### The Diagnostic Value of Three Ferroptosis-Related Biomarkers in Ischemic Stroke

**Figure 6:**
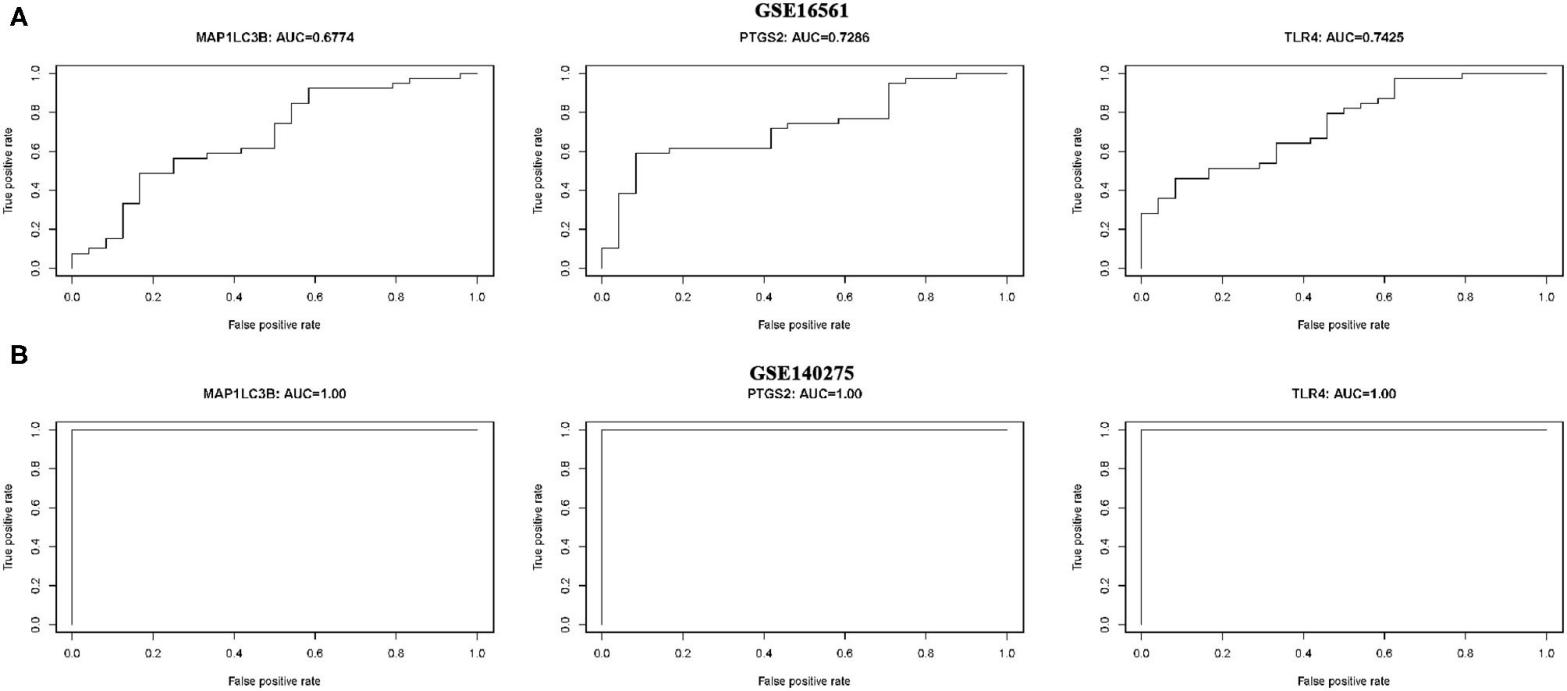
ROC monofactor analysis revealed the diagnostic value of ferroptosis-related biomarkers in ischemic stroke. **(A)** The AUC of ferroptosis-related biomarkers in the diagnosis of ischemic stroke in ROC monofactor analysis in the GSE16561 dataset. **(B)** The AUC of ferroptosis-related biomarkers in the diagnosis of ischemic stroke in ROC monofactor analysis in the GSE140275 dataset.

ROC monofactor analysis was performed in different datasets to evaluate the diagnostic value of three ferroptosis-related biomarkers in ischemic stroke. As a result, the accuracy of MAP1LC3B, PTGS2, and TLR4 in the diagnosis of ischemic stroke was 67.74, 72.86, and 74.25, respectively, in the GSE16561 dataset (Figure 6A). The ROC diagnostic accuracy of these three ferroptosis-related biomarkers for ischemic stroke were all 100% in the GSE140275 dataset (Figure 6B).

### Verification of Expression and Diagnostic Value of Three Ferroptosis-Related Biomarkers in Ischemic Stroke

**Figure 7:**
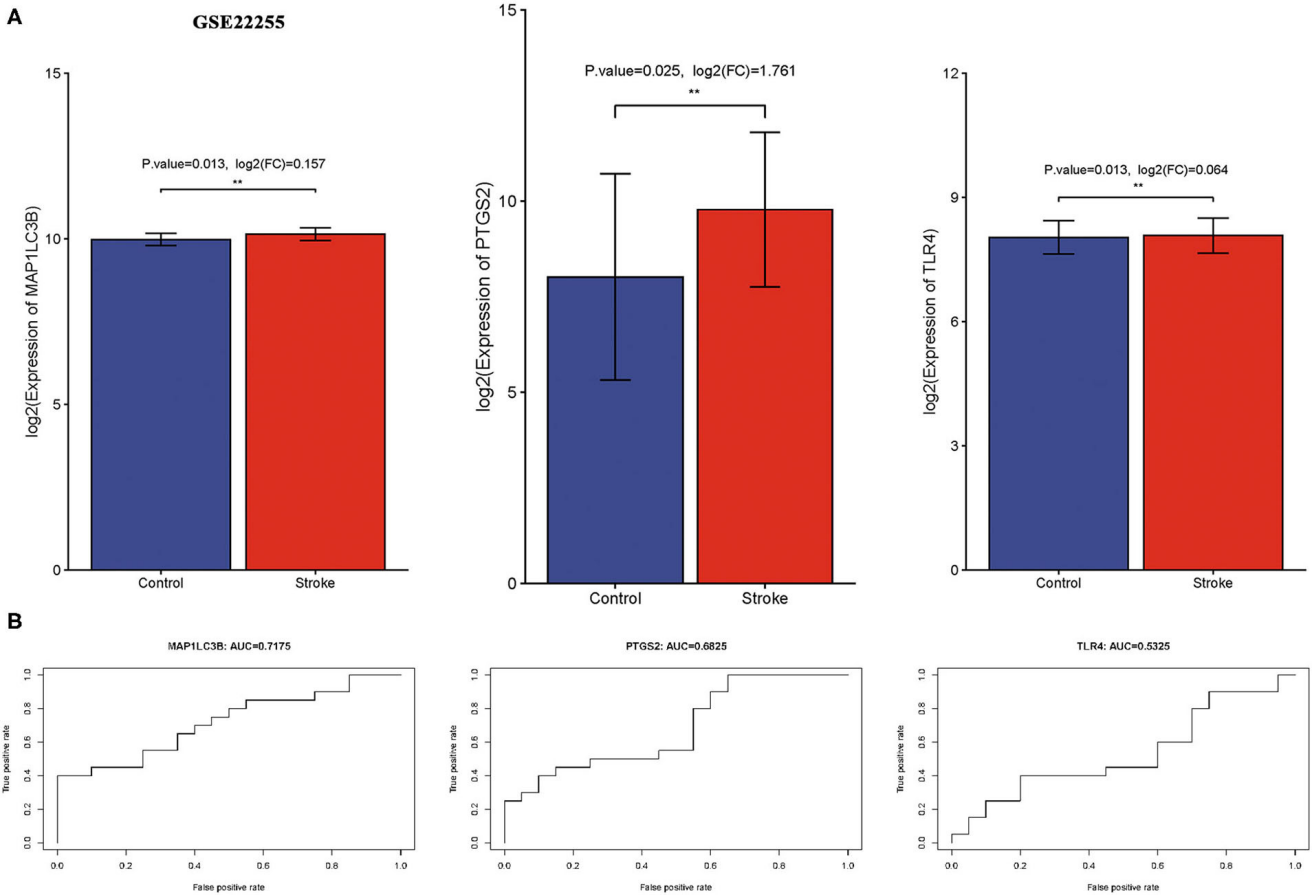
Verification of expression and diagnostic value of ferroptosis-related biomarkers in ischemic stroke. **(A)** The expression of ferroptosis-related biomarkers in ischemic stroke vs. normal control cohort in the GSE22255 dataset. **(B)** The AUC of ferroptosis-related biomarkers in the diagnosis of ischemic stroke in ROC monofactor analysis in the GSE22255 dataset. **p < 0.01.

We then verify expression and the diagnostic value of three ferroptosis-related biomarkers in ischemic stroke using the external data GSE22255. As expected, the data suggested that three ferroptosis-related biomarkers were also upregulated in the ischemic stroke vs. normal control, consistent with the result of the GSE16561 and GSE140275 datasets (Figure 7A, all *p* < 0.05). Moreover, the ROC diagnostic accuracy of MAP1LC3B, PTGS2, and TLR4 was 71.75, 68.26, and 53.25%, respectively (Figure 7B), further confirming the diagnostic value of three ferroptosis-related biomarkers in ischemic stroke.

### Common Transcription Factor Regulation Network

**Figure 8:**
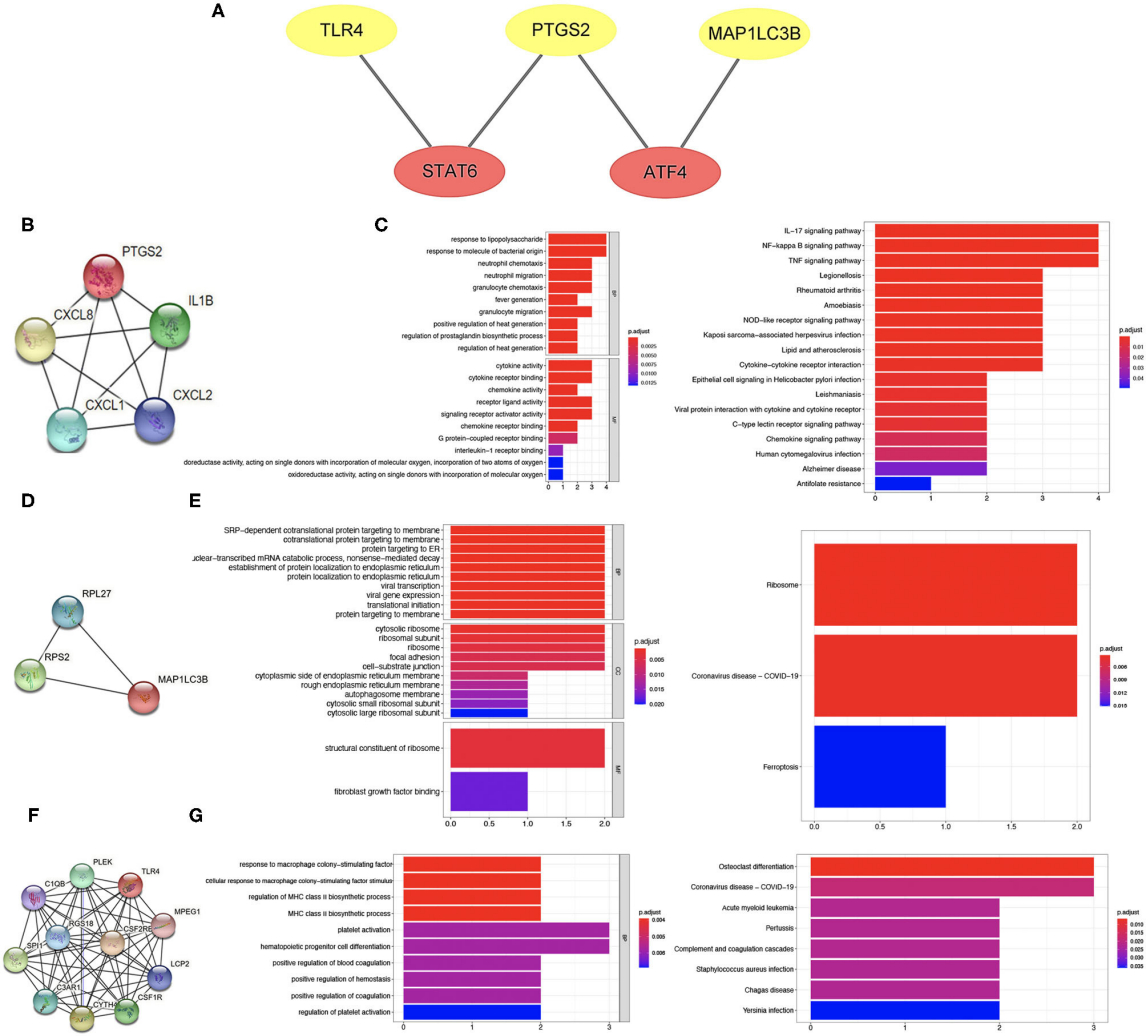
Transcription factor regulation network and co-expressed protein network of ferroptosis-related biomarkers. **(A)** Transcription factor regulation network of ferroptosis-related biomarkers. **(B,C)** Co-expressed protein network of PTGS2 and enriched items in GO and KEGG analysis. **(D,E)** Co-expressed protein network of MAP1LC3B and enriched items in GO and KEGG analysis. **(F,G)** Co-expressed protein network of TLR4 and enriched items in GO and KEGG analysis.

To further clarify the potential mechanism of ferroptosis-related biomarkers in ischemic stroke, we then explored the transcription factors of three ferroptosis-related biomarkers in ischemic stroke using the TRRUST v2 database. The regulation network was drawn with the core transcription factors that could regulate multiple ferroptosis-related biomarkers. The results showed that STAT6 and ATF4 were identified as the core transcription factors (Figure 8A). More specifically, STAT6 could regulate the expression of PTGS2 and TLR4, and ATF4 could regulate the expression of PTGS2 and MAP1LC3B, respectively (Figure 8A).

### Co-expressed Protein Network of Three Ferroptosis-Related Biomarkers

The co-expressed protein network was drawn using the proteins co-expressed with three ferroptosis-related biomarkers screened using the String database. Moreover, we also performed GO and KEGG analysis using the genes of co-expressed proteins. Four proteins were co-expressed with PTGS2 (Figure 8B), and the results of GO and KEGG analysis are shown in Figure 8C. The co-expressed protein network of MAP1LC3B and the functional enrichment map are shown in Figures 8D,E. Figure 8F shows the co-expressed protein of TLR4, suggesting that 10 proteins were co-expressed with TLR4. The results of GO and KEGG analysis based on these 10 proteins genes are shown in Figure 8G.

### Potential Therapeutic Compound Corresponding to Three Ferroptosis-Related Biomarkers for Ischemic Stroke

**Figure 9:**
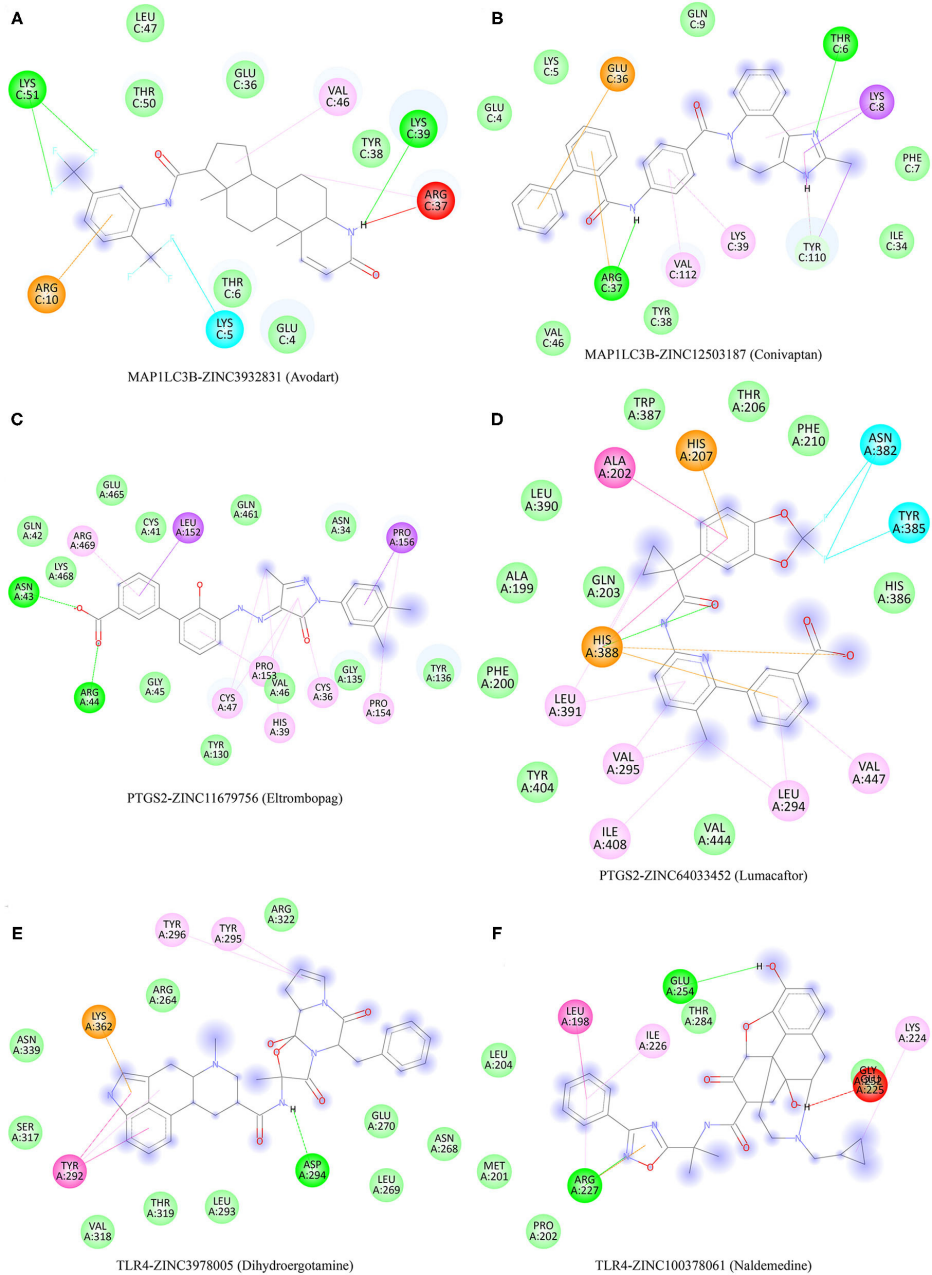
Potential therapeutic compounds corresponding to ferroptosis-related biomarkers for ischemic stroke. **(A,B)** Potential therapeutic compounds corresponding to MAP1LC3B for ischemic stroke. **(C,D)** Potential therapeutic compounds corresponding to PTGS2 for ischemic stroke. **(E,F)** Potential therapeutic compounds corresponding to TLR4 for ischemic stroke.

The protein structures of MAP1LC3B, PTGS2, and TLR4 were explored with the PDB database. A total of 2,115 FDA-approved small-molecule compounds were obtained from the ZINC15 database. These 2,115 small molecules were then molecularly matched with MAP1LC3B, PTGS2, and TLR4, respectively. Those compounds with the lowest binding energy were screened. As a result, the two small molecules with the lowest binding energy of MAP1LC3b were Zinc12503187 (Conivaptan) and Zinc3932831 (Avodart) (Figures 9A,B). The two small molecules with the lowest binding energy of PTGS2 were Zinc64033452 (Lumacaftor) and Zinc11679756 (Eltrombopag) (Figures 9C,D). Zinc100378061 (Naldemedine) and Zinc3978005 (Dihydroergotamine) were identified as the two small molecules with the highest affinity to TLR4 (Figures 9E,F).

## Discussion

Ferroptosis was a new form of programmed cell death linking metabolism, redox biology, and diseases (16). Accumulated evidence suggested that ferroptosis was involved in the pathological cell death related to degenerative diseases, carcinogenesis, stroke, and traumatic brain injury (16). A recent study revealed that ferroptosis plays a vital role in the progress of cerebral stroke (17). Clarifying the correlation between ferroptosis and ischemic stroke may provide novel biomarkers and ideas for the diagnosis and therapy of ischemic stroke.

We firstly explored the ferroptosis-related DEGs in ischemic stroke vs. normal control using GSE16561 and GSE140275. As a result, a total of 10 ferroptosis-related DEGs were identified. Moreover, GO and KEGG analysis revealed that these 10 ferroptosis-related DEGs were mainly enriched in response to oxidative stress, HIF-1 signaling pathway, NOD-like receptor signaling pathway, ferroptosis, lipid, and atherosclerosis. Interestingly, these pathways were involved in ischemic stroke. Wacker revealed that hypoxic preconditioning induces stroke tolerance *via* a cascading HIF and CCL2 signaling pathway (18). NLRP3, a member of NOD-like receptor signaling pathway, plays a vital role in the progression of ischemic stroke, suggesting NLRP3 as a potential treatment target for ischemic stroke (19, 20). Therefore, these 10 ferroptosis-related DEGs may also exert a significant role in ischemic stroke *via* these pathways.

By constructing a random forest model and performing ROC monofactor analysis, we identified three ferroptosis-related biomarkers, namely, MAP1LC3B, PTGS2, and TLR4, for ischemic stroke. These three ferroptosis-related biomarkers had a good performance in the diagnosis of ischemic stroke. Actually, a previous study had reported that MAP1LC3B, PTGS2, and TLR4 may play a vital role in ischemic stroke or served as biomarkers for ischemic stroke and other diseases. Another bioinformatic study revealed that TLR4 may be implicated in atrial fibrillation, a risk factor for ischemic stroke (21). TLR4 was a prognostic biomarker in breast cancer and associated with poor prognosis (22). Miao et al. found that smoking and drinking could affect the advancing of ischemic stroke *via* regulating PTGS2 and TNFAIP3 (23). Another study revealed that PTGS2 is a prognostic biomarker in colorectal cancer and correlated with high tumor recurrence risk and poorer specific survival (24). A previous study suggested that low MAP1LC3B expression was linked to poor prognosis in gastric cancer (25).

Another vital finding of our study is that several potential therapeutic compounds corresponding to MAP1LC3B, PTGS2, and TLR4 were also identified for ischemic stroke, including Zinc12503187 (Conivaptan), Zinc3932831 (Avodart), Zinc64033452 (Lumacaftor), Zinc11679756 (Eltrombopag), Zinc100378061 (Naldemedine), and Zinc3978005 (Dihydroergotamine). These were consistent with previous results. Conivaptan is an FDA-approved vasopressin receptor antagonist that may exert both osmotic and anti-inflammatory effects (26). Conivaptan could reduce brain edema and minimizes damage to the blood–brain barrier after stroke (27). Another study suggested that conivaptan could serve as a potential drug to reduce brain edema after stroke (28).

Our study also had some limitations. First, the ferroptosis-related genes included in our study may be incomplete. Moreover, it is desirable to verify our results using *in vivo* and *in vitro* experiments.

## Conclusion

All in all, our results suggested MAP1LC3B, PTGS2, and TLR4 as potential diagnostic biomarkers for ischemic stroke, providing more evidence about the vital role of ferroptosis in ischemic stroke.

## Data Availability Statement

The original contributions presented in the study are included in the article/supplementary material, further inquiries can be directed to the corresponding author/s.

## Author Contributions

GC performed data analysis work and aided in writing the manuscript. HT designed the study and assisted in writing the manuscript. LL edited the manuscript. All authors read and approved the final manuscript.

## Funding

This work was supported by the Zhejiang Provincial Natural Science Foundation of China (Grant No. LY19H090005) and the Science and Technology Project of Jinhua City (Grant No. 2018-3-025).

## Conflict of Interest

The authors declare that the research was conducted in the absence of any commercial or financial relationships that could be construed as a potential conflict of interest.

## Publisher's Note

All claims expressed in this article are solely those of the authors and do not necessarily represent those of their affiliated organizations, or those of the publisher, the editors and the reviewers. Any product that may be evaluated in this article, or claim that may be made by its manufacturer, is not guaranteed or endorsed by the publisher.

## Abbreviations

DEG: differentially expressed genes
GO: Gene Ontology
KEGG: Kyoto Encyclopedia of Genes and Genomes
BP: Biological processes
CC: Cellular components
MF: Molecular functions.
